# Analysis of IL-1β, CXCL8, and TNF-α levels in the crevicular fluid of patients with periodontitis or healthy implants

**DOI:** 10.1186/s12903-021-01478-3

**Published:** 2021-03-16

**Authors:** Paweł Aleksandrowicz, Ewa Brzezińska-Błaszczyk, Elżbieta Kozłowska, Paulina Żelechowska, Andrea Enrico Borgonovo, Justyna Agier

**Affiliations:** 1 Department of Periodontology, Medical University of Lublin, Lublin, Poland; 2 Department of Experimental Immunology, Medical University of Lodz, Lodz, Poland; 3 Department of Esthetic Dentistry, University of Milan, Milan, Italy

**Keywords:** CXCL8, IL-1β, Implants, Periodontitis, TNF-α

## Abstract

**Background:**

Our study aimed to assess the level of IL-1β, CXCL8, and TNF-α in peri-implant sulcular fluid (PISF) collected from patients with no clinical symptoms of mucositis or peri-implantitis and compare them with cytokine concentration in gingival crevicular fluid (GCF) acquired from patients with healthy periodontium and those with varying severity of periodontitis.

**Methods:**

A total of 189 subjects were included in the study, and GCF/PISF samples were checked for IL-1β, CXCL8, and TNF-α levels using an ELISA test.

**Results:**

The IL-1β level in PISF in patients with implants was significantly lower than in GCF in patients with mild, moderate, or severe periodontitis. The CXCL8 level in PISF was considerably lower than in patients with moderate periodontitis. The TNF-α level in PISF in patients with implants was markedly higher compared to subjects with healthy periodontium or patients with mild periodontitis.

**Conclusion:**

Analysis of cytokine levels may help describe the pathogenesis and early diagnosis of peri-implantitis and prevision in high-risk patients.

## Background

Over the last decades, dental implants have rapidly become an indispensable therapy in dentistry to replace one (or more) missing tooth. Although they have a high success rate, some of these interventions can end in failure. The most frequent dental implant complication is peri-implantitis, which occurs with a frequency ranging from 1 to 47% at the implant level [1, 2]. Peri-implantitis is an inflammatory response that affects the tissue surrounding the osseointegrated dental implant and results in excessive marginal bone loss [2]. Several limiting factors, such as poor oral hygiene, untreated periodontitis, untreated endodontic lesions, unfavorable osseous density, alcohol drinking, smoking, or diabetes, contribute to the therapy failure and induce peri-implantitis development and progression of inflammation. The implant's lifespan also depends on other elements such as quality of implant surface and appropriate attachment of connective tissue [3–5]. Peri-implantitis has been connected with a Gram-negative anaerobic microbiota, similar to that found in severe periodontitis around natural teeth [6, 7]. After implanting the implant, pathogenic bacteria migrate from periodontal pockets, tongue, tonsils, and inflamed gingiva to colonize the dental implant surface [8]. Bacterial dental plaque formation around dental implants leads to inflammatory reactions, which induce proliferation and an overgrowth of sulcular epithelium, the degeneration of connective tissue around the abutment, the loss of permucosal seal, and an epithelial migration [9, 10].

As in periodontitis, pathogens and their virulence stimulate the release of several immunoinflammatory biomarkers in peri-implant cells. The most meaningful mediators of inflammation are cytokines, which play an essential role in the pathogenesis of periodontal diseases and act as intermediaries in peri-implantitis [11]. The inflammatory process in regards to bacterial infection is mediated by the release of pro-inflammatory cytokines, such as interleukins (IL)-1β, IL-6, IL-12, and IL-17, tumor necrosis factor-alpha (TNF-α), chemokines CXCL8, and macrophage inflammatory protein (MIP)-1α, and neutrophil lysosomal enzymes, reactive oxygen species (ROS) or eicosanoids (prostaglandins, leukotrienes). Those mediators elicit tissue destruction and bone resorption by stimulating collagenase and the receptor activator of nuclear factor-kappa B ligand (RANKL), which induces osteoclast differentiation [11, 12]. Therefore, the evaluation of such cytokines level in the peri-implant sulcus fluid (PISF) has been suggested as a non-invasive method of monitoring the healthy or diseased states of the peri-implant tissues as well as the local response of peri-implant treatments [13, 14]. Using the cytokine assay in the analysis of PISF may help describe the pathogenesis stage more efficiently and predict an early diagnosis of peri-implantitis in high-risk patients. Despite investigative exertions to identify the levels of several cytokines in the PISF, the efficacy of these parameters to predict or contribute to the diagnosis of peri-implantitis is still undetermined.

Qualification of the state of periodontal and peri-implant tissues primarily depends on clinical checkups. The measurement of humoral factors' concentrations appearing during inflammation in gingival crevicular fluid (GCF) and PISF may be advantageous in assessing the early stage of periodontitis and/or peri-implantitis. Thus, this study aimed to investigate the IL-1β, CXCL8, and TNF-α levels in GCF in patients with different degrees of periodontitis and PISF in patients with healthy implants.

## Methods

### Patients study

The study group comprises 189 adult European Caucasian subjects: 85 men and 104 women between 20 and 71. Patients were selected and recruited from the Department of Periodontology at the Medical University of Lublin, and the study began on January 31, 2015, and ended on March 1, 2017. After a full explanation of this study's aim, written informed consent forms were obtained from all participants by the Helsinki Declaration. Medical and dental histories of each patient were gathered. Patient inclusion criteria were as follows: no treatment of periodontitis for the last 6 months that could affect periodontal treatment outcomes; no use of antibiotics and/or anti-inflammatory drugs for 3 months before taking part in the study. Criteria for exclusion from the study were as follows: pregnancy or breastfeeding for women. cigarette smoking, hormonal disorders or taking hormonal agents, infections (such as HIV, hepatitis, and tuberculosis), systemic diseases (e.g., diabetes, osteoporosis, and immunological disorders, and patients with a history of oncological treatment).

The diagnosis of patients was based on clinical and radiographic criteria. The clinical examination included Löe and Silness gingival index (GI), probing pocket depth (PD), clinical attachment level (CAL), and bleeding on probing (BOP). Both PD and CAL measurements were performed using a WHO-621 periodontal probe. GI measurements were assessed at four surfaces per tooth/implant (mesial, distal, buccal, lingual, and palatal surface), while PD and CAL measurements were taken at six surfaces per tooth (stepping method) (mesiobuccal, mid-buccal, distobuccal, mesio-oral, mid-oral and disto-oral). GI, PD, and CAL are given as average values. One examiner assessed all clinical parameters. Based on the clinical data, patients were later divided into five clinical groups as follow: (1) a control group of periodontally healthy patients (group I), (2) patients with mild periodontitis (group II), (3) patients with moderate periodontitis (group III) (4) patients with severe periodontitis (group IV), (5) periodontally healthy subjects who received an implant treatment (implants with a new alternative hydrophilic surface SPI ELEMENT INICELL, Thommen Medical AG, Grenchen, Switzerland and Brånemark system implant, Nobel Biocare, Gothenburg, Sweden) (group V). In the study group of patients who have received dental implants, none showed any symptoms of peri-implantitis and had no prior history of periodontitis. The intraoral periapical radiographic method was used to evaluate each implant's bone condition and was obtained for each implant using the paralleling technique with a radiographic positioner. In the qualification, we also considered the type of prosthetic work performed on implants. In all patients from group V, the bone density grade was determined according to a scale of D1-D4 defined by Misch [15].

### GCF/PISF sampling and processing

Before a GCF sampling, the supragingival plaque was carefully removed. The collection sites were isolated using cotton rolls and dried with air jets. The GCF samples were subsequently obtained from the mesiobuccal root using sterile Periopaper strips (Oraflow Inc., Plainview, NY, USA) that were overlaid and placed at the gingival crevice region until mild resistance was felt. The strips were left in place for 30 s to prevent any mechanical irritation. The strips contaminated with blood were discarded. Following the GCF collection, the strips were kept in sterile test tubes and stored in aliquots at − 80 °C until needed for analysis.

Clinical examinations in the group of patients with implants were performed after removal of the supra-constructions. PISF samples have been drawn at least 18 months after the surgery, similarly, using sterile Periopaper strips that were inserted into the gingival crevice until mild resistance was felt. For 30 s, the strips were left in place. After that, the paper points were transferred to a sterile test tube and then immediately stored in aliquots at a temperature of − 80 °C [16].

### Cytokine measurements

For GCF/PISF extraction, paper strips were put in tubes containing 500 µL of phosphate-buffered saline (PBS) (pH 7.2) (Sigma Aldrich) and next gently shaken and incubated at room temperature for 1 h. After that, the strips were pulled, and the fluids were analyzed. Commercially available enzyme-linked immunosorbent assays (ELISA) were used to measure the concentration of IL-1β (catalog no DLB50), CXCL8 (catalog no D8000C), and TNF-α (catalog no DTA00C) (Quantikine R&D Systems Inc., Minneapolis, MN, USA) on Thermo Scientific™ Multiskan™ FC Microplate Photometer. All ELISA procedures were performed according to the manufacturer's instructions. All measurements were done in triplicate. The GCF/PISF IL-1β, CXCL8, and TNF-α concentrations were equated to a standard calibration curve.

### Statistical analysis

The statistical analysis for this study was conducted using Statistica 13.1 (Statsoft Inc., USA). Shapiro–Wilk test was used to analyze the normality of distribution, while the Mann–Whitney U test was performed to analyzed differences in the levels of IL-1β, CXCL8, and TNF-α in GCF, and differences in the levels of IL-1β, CXCL8, and TNF-α between G1 and G2 bone density groups, as well. Associations between studied proteins were tested using linear regression (adjusted for age and sex). The Spearman's rank correlation coefficient was used to test correlations between IL-1β, CXCL8, and TNF-α concentrations in G1 and G2 bone density groups. Statistical significance was set at *p* = 0.05.

## Results

The baseline characteristics of each study group are presented in Table 1. The control group of periodontally healthy patients consisted of 36 subjects (13 men and 23 women, aged 35 ± 8 years) with no clinical evidence of gingival inflammation, no radiographic evidence of alveolar bone loss, no tooth loss due to periodontitis, PD < 3 mm, CAL 0–4.5 mm, BOP < 10%; Group II consisted of 48 patients with mild periodontitis (18 men and 30 women, aged 38 ± 9 years) with PD 3–4 mm, CAL 0–4.5 mm, BOP > 10%, radiographic bone loss-coronal third < 15%, no tooth loss due to periodontitis. Group III consisted of 43 subjects with moderate periodontitis (21 men and 22 women, aged 40 ± 9 years) with PD 4–6 mm, CAL 0–6 mm, 10% < BOP < 50%, radiographic bone loss-coronal third from 15 to 33%, tooth loss due to periodontitis of ≤ 4 teeth. Group IV consisted of 30 patients with severe periodontitis (18 men and 12 women, aged 42 ± 10 years) with PD > 6 mm, CAL 1.5–10 mm, BOP ≥ 50%, radiographic bone loss-extending to mid-third of root and beyond, tooth loss due to periodontitis ≥ 5 teeth. Group 5 consisted of 32 periodontally healthy subjects (15 men and 17 women) who received an implant treatment. The patients received a single tooth implant (15 subjects) or multiple units (17 subjects). The lifespan of a dental implant ranges from 36 to 147 months (Table 2). The patients enrolled in the study did not have any bone changes in the area of the implants.

**Table 1:** Demographic and clinical characteristics of study population

	Control (I)	Mild periodontitis (II)	Moderate periodontitis (III)	Severe periodontitis (IV)	Implant (V)
*N*	36	48	43	30	32
*Age (years)*					
Mean ± SD	35 ± 8	38 ± 9	40 ± 9	42 ± 10	52 ± 16
Range	20–51	27–58	27–60	28–59	20–71
*Gender*					
Male	13	18	21	18	15
Female	23	30	22	12	17
*Total natural teeth*					
Mean ± SD	29 ± 3	28 ± 3	27 ± 3	26 ± 3	14 ± 11
Range	24–32	20–32	22–32	22–32	0–29
*Total implants*					
Mean ± SD	0	0	0	0	6 ± 3
Range	–	–	–	–	1–12
*PD (mm)*					
Mean ± SD	1.64 ± 0.61	3.34 ± 0.39	4.49 ± 0.23	5.55 ± 0.42	2.84 ± 0.57
Range	0.2–2.5	3–4	4–5	4–6	1.9–4
*CAL (mm)*					
Mean ± SD	0.71 ± 1.20	1.14 ± 1.32	1.80 ± 1.60	6.48 ± 1.65	–
Range	0–4.5	0–4.5	0–6	1.5–10	
*GI (mm)*					
Mean ± SD	0.81 ± 0.98	1.27 ± 0.74	1.30 ± 0.6	1.67 ± 0.71	0.34 ± 0.55
Range	0–3	0–2	0–2	0–3	0–2

SD, standard deviation

**Table 2:** Characteristic of patients with implants and prosthodontics restoration

Type of restoration	Patient	Examined sulcus implant position	Functioning period of restoration (months)	Bone density
Implant crown	1	11	56	D3
	2	13	55	D3
	3	23	102	D4
	4	11	58	D3
	5	24	47	D3
	6	44	83	D3
	7	34	59	D3
	8	14	36	D3
	9	14	46	D4
	10	34	46	D2
	11	24	45	D4
	12	14	45	D4
	13	46	43	D2
	14	36	79	D2
	15	46	59	D2
Multiple units	16	24	55	D2
	17	43	83	D1
	18	43	84	D2
	19	23	48	D3
	20	14	42	D2
	21	25	45	D3
	22	15	74	D3
	23	24	49	D3
	24	42	49	D1
	25	23	38	D3
	26	34	147	D4
	27	44	39	D1
	28	23	48	D3
	29	44	48	D2
	30	15	79	D3
	31	23	114	D4
	32	43	114	D1

Right side of maxilla 1; Left side of maxilla 2; Right side of mandibula 3; Left side of mandibula 4; 1: incisal; 2: incisal; 3: canine; 4: first premolar; 5: second premolar; 6: first molar. /FDI International Status Tooth Identification/

IL-1β, CXCL8, and TNF-α levels in GCF/PISF in patients with healthy periodontium, patients with varying severity of periodontitis, and implants are presented in Table 3. As expected, we found that the mean concentration of IL-1β was the highest in patients with severe periodontitis (61.04 ± 41.41 pg/mL). IL-1β level in PISF in patients with implants reached 23.73 ± 27.07 pg/mL and was significantly lower than in GCF in patients with mild (*p* = 0.029), moderate (*p* = 0.0005), and severe (*p* = 0.000014) periodontitis. The statistical analysis also showed that the GCF concentration of IL-1β was greater in patients with mild (*p* = 0.0008), moderate (*p* = 0.000003), and severe (*p* = 0.0000001) periodontitis compared to patients with healthy periodontium, and higher in patients with severe than mild periodontitis (*p* = 0.003).

**Table 3:** The comparison of IL-1β, CXCL8, and TNF-α levels in patients’ groups

	Control (I; n = 36)	Mild periodontitis (II; n = 48)	Moderate periodontitis (III; n = 43)	Severe periodontitis (IV; n = 30)	Implant (V; n = 32)
*IL-1β (pg/mL)*					
Mean ± SD	16.90 ± 18.65	36.16 ± 33.98	46.76 ± 38.62	61.04 ± 41.41	23.73 ± 27.07
Range	0.10–77.90	0.10–156.80	0.10–181.90	6.40–153.80	0.10–125.70
*CXCL8 (pg/mL)*					
Mean ± SD	37.91 ± 34.06	44.10 ± 38.05	58.16 ± 55.26	51.94 ± 45.87	40.90 ± 56.63
Range	3.20–132.20	0.90–205.20	4.90–274.30	2.50–185.20	3.10–296.30
*TNF-α (pg/mL)*					
Mean ± SD	4.31 ± 2.02	4.56 ± 2.15	5.41 ± 2.12	5.34 ± 2.39	5.71 ± 1.94
Range	0.10–9.10	0.10–10.20	1.30–9.50	1.20–11.20	1.50–9.40

The Mann–Whitney U test was performed to analyzed differences in the levels of IL-1β, CXCL8, and TNF-α in GCF/PISF. Statistical significance was set at *p* = 0.05

It was observed that the concentration of CXCL8 in each group of patients with periodontitis varied significantly according to the stage of the disease. The CXCL8 level in PISF was similar to the CXCL8 level in GCF in healthy patients and ranged from 3.1 to 296.3 pg/mL with a mean of 40.90 ± 56.63 pg/mL. Statistical analysis revealed that the CXCL8 level in PISF was significantly lower than in patients with moderate periodontitis (*p* = 0.011). Furthermore, in patients with moderate periodontitis, CXCL8 levels in GCF were statistically higher than in periodontally healthy subjects (*p* = 0.046).

TNF-α’s mean concentration was the highest in patients with moderate periodontitis (5.41 ± 2.12 pg/mL) and significantly greater than in GCF of healthy subjects (*p* = 0.025). TNF-α level in PISF in patients with implants reached 5.71 ± 1.94 pg/mL and was markedly higher compared to subjects with healthy periodontium (*p* = 0.003) and patients with mild periodontitis (*p* = 0.010). Additionally, the statistical analysis showed no differences between the level of the studied cytokines/chemokines evaluated in terms of age, sex, or gingival index.

We have also evaluated whether there was any relationship between cytokine levels in PISF and the bone density grade (patients with bone density D1 and D2 formed group G1, n = 12; patients with bone density D3 and D4 formed group G2, n = 20); detailed characteristics of these groups are presented in Table 4. Statistical analysis showed no significant differences between G1 and G2 groups for any of the cytokine/chemokine levels (IL-1β *p* > 0.05; CXCL8 *p* > 0.05; TNF-α *p* > 0.05). A statistically significant positive correlation between IL-1β and CXCL8 levels in PISF was observed in the G1 group (r = 0.6827; *p* = 0.014). Linear regression adjusted for age and sex revealed no association between studied protein levels in the study groups.

**Table 4:** IL-1β, CXCL8, and TNF-α levels in PISF depending on bone density

	G1 (bone density D1 + D2)	G2 (bone density D3 + D4)
*IL-1β (pg/mL)*		
Range	1.00–125.70	0.10–63.20
Mean ± SD	35.44 ± 36.11	16.70 ± 17.40
*CXCL8 (pg/mL)*		
Range	4.90–107.70	3.10–296.30
Mean ± SD	33.99 ± 30.94	45.06 ± 68.04
*TNF-α (pg/mL)*		
Range	1.50–9.40	3.10–9.40
Mean ± SD	5.39 ± 2.41	5.91 ± 1.64

SD, standard deviation

The Mann–Whitney U test was performed to analyzed differences in the levels of IL-1β, CXCL8, and TNF between G1 and G2 bone density groups. The Spearman's rank correlation coefficient was used to test correlations between IL-1β, CXCL8, and TNF-α concentrations in G1 and G2 bone density groups. Statistical significance was set at *p* = 0.05

## Discussion

Few of the available scientific literature data suggest that the levels of inflammatory mediators in GCF and PISF, including cytokines, chemokines, and matrix metalloproteinases (MMPs), may be used to assess the periodontal status of peri-implant tissues and to monitor the development of inflammation. The results presented in these publications are inconclusive, so in this study, we decided to evaluate the levels of IL-1β, CXCL8, and TNF-α in PISF obtained from patients without clinical symptoms of mucositis or peri-implantitis and compared them with the levels of mediators in GCF obtained from patients with healthy periodontitis and with varying degrees of periodontitis. In the presented study, we showed that the level of IL-1β in PISF in patients with implants was significantly lower than in GCF in patients with mild, moderate, or severe periodontitis. In contrast, the level of CXCL8 in PISF was substantially lower than in patients with moderate periodontitis. Moreover, the level of TNF-α in PISF in patients with implants was significantly higher than in those with healthy periodontitis or mild periodontitis.

IL-1β is a multifunctional cytokine with diverse biologic activities implicated within the pathophysiology of periodontitis and peri-implantitis. IL-1β is the most crucial cytokine that stimulates bone resorption of the alveolar process. It increases the expression of collagenolytic enzymes, MMPs, which contribute to extracellular matrix degradation and, in turn, bone resorption and tissue destruction [17, 18]. Moreover, IL-1β strongly initiates inflammatory processes. In the following study, we found that the mean level of IL-1β was the highest in patients with severe periodontitis. Similar observations and conclusions were made by Ramseier et al. [19]. Their research work showed that IL-1β levels vary in GCF depending on periodontal conditions, with the highest concentration in mild to moderate periodontitis. Similar results were presented by Gonzales et al. [20], who found and described that the level of IL-1β in GCF increases with the severity of clinical symptoms of gingivitis. Heasman et al. [21] found that IL-1β levels in GCF during the development of experimental gingivitis increased 8-times, compared to baseline, one week after the primary measurement. Rawlinson et al. [22] also showed a robust relationship between the severity of inflammation within the periodontium and IL-1β in GCF. Interesting studies were conducted by Engebretson et al. [23]. The authors found that the level of IL-1β in GCF increases with the progression of the periodontitis, and in patients with the most severe form of the disease, it is almost 8 times higher than in the control group. Our study showed that the level of IL-1β in PISF among patients with healthy implants was significantly lower than in GCF in patients with all stages of periodontitis. Yaghobee et al. [24] observed and described a remarkable difference between the level of IL-1β in GCF from the gingiva around the natural tooth (45.71 pg/μL) and PISF from the peri-implant tissue (75.26 pg/μL). In turn, Nowzari et al. [25], Recker et al. [26], and Teixeira et al. [27] demonstrated and described a comparable level of this cytokine between GCF and PISF in healthy volunteers. Güncü et al. [28] observed a significantly higher level of IL-1β in patients with gingivitis/inflamed dental implants than in healthy patients without inflammation around the implant. Interestingly, Abduljabbar et al. [29] research showed that the level of IL-1β in PISF is statistically significantly higher among individuals smoking waterpipe compared with non-smokers. Moreover, Renvert et al. [30] observed that in the presence of profuse bleeding and with a probing depth ≥ 6 mm, higher levels of IL-1β in PISF were found than in material from implant sites with minor bleeding on probing, no suppuration, and with a probing depth ≤ 5 mm.

Because CXCL8 is a potent chemoattractant cytokine and activator of neutrophils in inflammatory regions released from gingival fibroblasts in the gingival crevice, we have also assessed the level of this cytokine. The available literature data on the level of CXCL8 in GCF regarding the clinical condition are inconclusive and often even contradictory. Jin et al. [31] found lower CXCL8 levels in GCF in patients with periodontitis than healthy people. In turn, Chung et al. [32], when comparing CXCL8 concentration in GCF in periodontologically healthy people and patients with periodontitis, observed a significantly higher level of chemokine in people with healthy periodontium. In our studies, the CXCL8 level in PISF was similar to the CXCL8 level in GCF in healthy patients.

Furthermore, we have found that the CXCL8 level in PISF was significantly lower than in GCF from patients with moderate periodontitis. Our results confirmed the observations of Recker et al. [25], Severino et al. [33], and Ata-Ali et al. [34]. In turn, Hall et al. [35] and Lagdive et al. [36] observed that the level of CXCL8 was significantly upregulated in the peri-implantitis probes. In turn, Renvert et al. [30] showed higher levels of CXCL8 in PISF in the presence of profuse bleeding than in material from implant sites with minor bleeding on probing. It seems that the assessment of CXCL8 levels in GCF or PISF cannot be a measurable indicator of the dynamics of the inflammatory process in periodontal tissues. This is most likely due to the fact that CXCL8 is present in GCF, even in people without any inflammatory changes within the periodontal tissues [37–39]. It is perhaps related to the defense process of "physiological" inflammation in the gingival gap, where neutrophils play a significant role.

TNF-α is considered to be a crucial cytokine involved with the innate response against the periodontopathogenic bacteria. Even though no correlation can be observed between the levels of TNF-α in GCF in different degrees of periodontitis, this molecule showed a strong correlation with the severity of periodontal destruction, and it could be used to compare the various stages of periodontal disease [40]. We have established that TNF-α concentration was remarkably higher in patients with implants than subjects with healthy periodontium and patients with mild periodontitis. Significantly higher TNF-α levels were also noted in PISF compared with their levels in GCF by Recker et al. [26]. Interestingly, Renvert et al. [30] stated that profuse bleeding and/or suppuration in untreated peri-implantitis could be associated with higher concentrations of TNF-α in PISF. The biological processes occurring in bone structures after implantation are the result of both resorption and osteogenesis. The osseointegration process between the implant surface and the surrounding bone tissue takes place in several stages, and its last phase is the internal bone reconstruction, called bone remodeling. Bone remodeling is an active and dynamic process that relies on the correct balance between bone resorption by osteoclasts and osteoblasts' bone deposition.

These two functions need to be closely related not only in terms of quantity but also in time and space [41, 42]. It should be emphasized that this continuous bone remodeling process ensures the long-term functionality of the implant. Bone turnover processes are regulated by various humoral factors, including a significant role of TNF-α. Undoubtedly, TNF-α is a multidirectional cytokine that exerts many different effects, but it is mostly known for its vigorous pro-inflammatory activity. Of note, TNF-α is also involved in the regulation of bone remodeling [43, 44]. Hence, increased TNF-α levels in PISF from patients with implants may constitute an indicator of ongoing remodeling processes. In turn, elevated TNF-α levels may also suggest the initiation of an inflammatory process within the tissues around the implant with no clinical symptoms.

It should be noted that the beginning of peri-implantitis is asymptomatic; thus, our results might suggest that the monitoring of cytokine levels in PISF could support identify peri-implantitis in an early stage, before clinical manifestations. Additionally, in our previous research, we have observed that scaling and root planing (SRP) in patients with chronic periodontitis led to a significant decrease in MMP-8 concentration in GCF [45] and that the level of this factor in PISF received from the patients without any signs of peri-implantitis was notably higher than in GCF of periodontally healthy patients [16].

## Conclusion

In conclusion, our observation might imply that the monitoring of TNF-α, CXCL8, and IL-1β levels in GCF and PISF can be considered as one of the diagnostic elements. Analysis of cytokine levels may help describe the pathogenesis and early diagnosis of peri-implantitis and prevision in high-risk patients. Especially, increased levels of TNF-α in PISF in patients with healthy implants may be an indicator of the ongoing process of tissue remodeling around the implant. Elevated TNF-α levels may also suggest the initiation of an inflammatory process within the tissues around the implant with no clinical symptoms. Nonetheless, our study has some limitations in the context in which our findings need to be interpreted carefully. Firstly, in this study, there were a relatively low number of subjects in each group. Secondly, there are only a few prior research studies on the issue. Finally, it was a cross-sectional study; therefore, we cannot compare the studied proteins' concentration in patients before inserting the implant.

## Data Availability

The data that support the findings of this study are available from the corresponding author, J. Agier, upon reasonable request.

## Abbreviations

BOP: Bleeding on probing
CAL: Clinical attachment level
GCF: Gingival crevicular fluid
GI: Gingival index
MIP-1α: Macrophage inflammatory protein
PISF: Peri-implant sulcular fluid
PD: Probing pocket depth
ROS: Reactive oxygen species
TNF-α: Tumor necrosis factor-alpha
